# Patient’s access to healthcare and treatment in rheumatoid arthritis: the views of stakeholders in Portugal

**DOI:** 10.1186/1471-2474-14-279

**Published:** 2013-09-25

**Authors:** Pedro A Laires, Rui Mesquita, Luís Veloso, Ana Paula Martins, Rui Cernadas, João Eurico Fonseca

**Affiliations:** 1 Merck Sharp & Dohme, Outcomes Research, Quinta da Fonte, Edificio Vasco da Gama, nº19, Paço de Arcos 2770-192, Portugal; 2 Merck Sharp & Dohme, Medical Affairs, Oeiras, Portugal; 3 Eurotrials, Consultores Científicos, Lisbon, Portugal; 4 Administração Regional de Saúde (ARS) Norte, Oporto, Portugal; 5 Lisbon Academic Medical Centre, Lisbon, Portugal

**Keywords:** Rheumatoid arthritis, Biologics, Access to treatment, Qualitative research, Stakeholders

## Abstract

**Background:**

The access to healthcare and treatment by rheumatoid arthritis (RA) patients, particularly to biologics, differs significantly among European countries.

We aimed to explore the views and experiences of Portuguese healthcare stakeholders on key barriers which limit the access to treatment, and ultimately to biologics, by RA patients and to find potential solutions (leverage points) to overcome the identified barriers.

**Methods:**

This was a qualitative research consisting of semi-structured face-to-face interviews with key stakeholders in RA framework. Thirty four individuals from eight groups of stakeholders were interviewed: rural and urban general practitioners (GPs), rheumatologists, hospital managers, hospital pharmacists, budget holders, representatives from the Portuguese Rheumatology Society and the RA Patient Association. Interviews were conducted between May and June 2011. Conventional content analysis with research triangulation was used.

**Results:**

The key barriers identified were related to the accessibility to primary healthcare services, difficulties in RA diagnosis among GPs, inefficient referral to secondary healthcare and controlled process of biologics prescription in public hospitals. The leverage points identified included the improvement of epidemiological and clinical knowledge about RA in Portugal, a better understanding of the disease among patients and GPs, the clarification of biologics benefits among budget holders and a raised awareness of the current treatment guidelines. In order to further address the leverage points, the following key initiatives were proposed: optimization of RA national registry; dissemination of information on rheumatic symptoms in primary care facilities and among the general public; increase interaction between rheumatologists and GPs through clinical discussions of successfully treated patients or workshops; broader utilization of disease diagnosis and monitoring tools, such as DAS28, and implementation of hospital–based research to collect real-world data.

**Conclusions:**

Most of the key barriers limiting the access to treatment, including biologics, in RA in Portugal are upstream of rheumatology practice. Our findings suggest that future actions should be focused on the primary care level to improve referral to rheumatologists. In addition, the collection of real-world data seems essential to characterise the RA population, to improve disease management and to increase compliance with current treatment guidelines.

## Background

Rheumatoid arthritis (RA) is a chronic systemic inflammatory disease, which primarily causes a symmetric polyarthritis, clinically manifested by joint pain, stiffness, and swelling [1]. It is estimated to affect between 0.5 and 1.0% of the adult population in Western countries [2]. Untreated, most patients have a progressive course, resulting in short- and long-term disability [3]. Evidence shows that an early diagnosis and management of RA can improve disease outcome [4-6]. The European League Against Rheumatism (EULAR) recommends that patients presenting with arthritis should be referred to and seen by a rheumatologist, ideally within six weeks after the onset of symptoms [7]. However, several studies have shown a delayed access to the treatment by RA patients at different settings [8-11].

The introduction of biologics in the late 1990s revolutionized the management of RA. Despite its widespread availability there are differences in the biologic drugs use across Europe. Based on IMS sales data and adjusted estimates of RA prevalence in Europe Kobelt et al. estimated a variation of biologics use ranging from 1% to 30% of the diagnosed adult patient population in 2008. In Portugal, the observed proportion was 5.5%, the second lowest among the smallest western markets in Europe. Different factors can explain these variations. The two most important are the macro-economic environment and the treatment guidelines. The limited use of biologics can be a consequence of low access to specialists and the cost of biologics, the later contributing to restrictive treatment guidelines and administrative constrains, most likely related to GDP in each country [12]. These findings led Laires et al. to conduct a desk-based research to compare the proportion of RA patients treated with biologics across Europe and to investigate the factors that most influence its use, with focus on the Portuguese case. In 2010, less than 7% of the estimated RA patient population was using biologics in Portugal, which is noticeably lower than the average rate of selected European countries (19%). This investigation also suggested that countries with the highest levels of consumption of biologics showed superior methotrexate consumption, higher GDP per capita and biologics retail distribution [13].

A desk research complemented by semi-structured interviews to senior physicians and patient representatives in France, Germany, Italy, Spain and UK, found that the number of barriers conditioning the access to treatment in RA varied considerably between those countries. The main limiting factors included primarily delays in the diagnosis of RA due to the lack of specialists available and limited RA expertise among general practitioners (GPs), resulting in slow referrals to the rheumatologists. In addition, a low RA awareness in the population, contributes to a late consultation with the GP, worsening the global scenario. The access to biologics by patients with a pre-existing RA diagnosis is limited by differences in the reimbursement and prescription process and variations in the strictness of the treatment guidelines across countries [14].

It is therefore pertinent to explore the views and experiences of Portuguese stakeholders directly involved in the RA therapeutic circuit and to identify the main barriers to access treatment, and ultimately to biologics, in order to improve this situation. To our knowledge, no such research has been conducted in Portugal before. This article, draws out the perspectives of its participants, presents qualitative findings regarding the key factors conditioning RA patient’s access to specialized healthcare and appropriate treatment and identifies potential solutions (leverage points).

## Methods

In order to better understand the main barriers and possible leverage points a qualitative research was performed using a semi-structured interview with key stakeholders in Portugal. The interviews were conducted by researchers qualified in qualitative research methods and independent reviewers analysed the data.

### Selection of stakeholders

The stakeholders were purposely selected based on their involvement in the RA therapeutic circuit. Candidates from eight groups of stakeholders were invited to take part: GPs from urban and rural areas, rheumatologists, hospital pharmacy managers and purchasers; hospital managers; regional and national budget holders, representatives from the Portuguese Society of Rheumatology (SPR) and National RA Patient Association (ANDAR). Physicians and hospitals from the most relevant geographical areas were selected. There was no predefined number of interviews per stakeholder group. The interviews to each group of stakeholders continued until overall data saturation was achieved, i.e. when no new themes emerged and the same themes were reiterated.

### Preliminary desk research

Preliminary desk research based on consultation of an expert panel and relevant literature review [12] yielded valuable information on the RA framework. From this preliminary work an interview script was developed with five pre-determined major research topics and related questions: 1) Estimation of target RA patients; 2) Access to healthcare services; 3) Access to RA treatment; 4) Treatment with biologics; 5) Hospital financing/management. These topics allow the characterisation of the access to healthcare and treatment by RA patients. The complete list of questions is displayed in Additional file 1.

Open questions were formulated for each topic to explore the views of stakeholders on potential barriers, leverage points and specific initiatives.

### Interviews to the stakeholders

Face to face interviews were carried out between May and June 2011. Upon the explanation of the research objectives by the interviewer a written informed consent for data collection and publication of anonymous data was obtained from all participants.

A trained and qualified interviewer followed a script tailored to the background of the stakeholders with questions covering the major research topics. Stakeholders were encouraged to openly express their points of view. Both the identified barriers, leverage points and set of initiatives that could contribute to overcome the barriers were debated between the interviewer and each stakeholder. Each initiative was graded according to the following criteria: attractiveness to stakeholders (not interesting, likely interesting and clearly interesting) and expected impact of the initiative in overcoming the identified barriers (low, medium and high).

Each interview lasted approximately 60 minutes and was audio taped and later transcribed. In addition, interviewer’s field notes documented the contextual details and the non-verbal expressions of the interviewees, which were useful in the data analysis and interpretation. The interviewees were blinded about the funding of this project.

### Qualitative analysis

Conventional content analysis was used through immersion/crystallization cycles as well as grounded theory approaches. The interviews were transcribed verbatim and organised thematically by the interviewer. Two independent researchers were involved in all the analysis steps. First, the data was read to achieve a good knowledge of the content. Then, portions of data that were similar in concept or idea were coded. For example, the ideas related to the inefficient referral from primary healthcare services or the waiting times at the hospital were coded as “difficulties in accessing secondary healthcare services”. The concepts were compared between transcripts and documents and in case of inconsistencies data portions were re-coded. The codification and concepts were also discussed between the researchers in order to achieve consensus. Afterwards, the researchers categorised each barrier identified under one of the five major topics described (see Table 1). Each category was associated with specific leverage points and initiatives. Based on the content of the interviews the researchers graded (as low, medium or high) each key initiative regarding the investment and effort required. No qualitative analysis software was used to manage the data.

**Table 1:** Major research topics addressed by each group of stakeholders

		**Urban GPs**	**Rural GPs**	**Rheumatologists**	Hospital pharmacy managers and purchasers*	**Hospital managers**	**Budget holders**^**†**^	**SPR**	**ANDAR**
**Number of interviews**		**5**	**5**	**9**	**6**	**4**	**3**	**1**	**1**
**Major research topics**	Estimation of target RA patients			•				•	•
	Access to healthcare services	•	•	•				•	•
	Access to RA treatment			•	•	•		•	•
	Treatment with biologics			•	•	•		•	•
	Hospital financing/management			•	•	•	•	•	•

*Directors responsible for hospitals’ purchasing departments. ^†^Regional and national budget holders from Central Administration of Health System (ACSS).

GPs: General practitioners, RA: Rheumatoid Arthritis, SPR: Portuguese Society of Rheumatology, ANDAR: National Rheumatoid Arthritis Patient Association.

In order to confer more robustness, the main conclusions from the interviews, including the classification attributed to each initiative, were consolidated in a moderated group discussion involving one interviewee from each group of stakeholders.

Qualitative research exploring the viewpoints and opinions of healthcare professionals does not require ethical approval in Portugal.

## Results

There were thirty four participants in the project. Each group of key stakeholders contributed with data from one to nine individuals. All groups of stakeholders were represented as planned (see Table 1).

Four key barriers limiting access to treatment, including biologics, were identified as a result of the interviews with stakeholders (see Table 2).

a) Difficulties in accessing primary healthcare services

The reduced proximity to primary healthcare centres in certain rural areas was seen by the GPs as an important accessibility constrain. The lack of primary healthcare physicians was considered another relevant limitation in these regions. However, these aspects were not valued by urban GPs. Further, GPs believe there is a lack of information regarding RA among the population, which is often mistaken with other pathologies.

b) Difficulties in RA diagnosis among GPs

According to the rheumatologists, SPR and ANDAR’s perspective, RA can be easily confused with other rheumatic diseases and therefore difficult to diagnose during the first appointments. In addition, the time to perform complementary tests is usually long, delaying the diagnosis. Due to unawareness of its advantages or lack of funding for it, the anti-cyclic citrullinated peptide (CCP) test is not performed routinely in primary care. Anti-CCP is a widely recognised tool for RA diagnosis and very useful for non-rheumatologists who are not experienced in joint examination [7]. GPs believe that the availability of these tests would improve the referral of RA patients to the specialist.

c) Difficulties in accessing secondary healthcare services

The group of professionals interviewed regarding this topic, which included GPs, rheumatologists, SPR, and ANDAR, referred that the waiting period for a consultation with the specialist can vary between regions. The access to a specialist is more difficult in rural areas, as hospitals are distant, with a deficient public transport network. From the rheumatologists and SPR point of view, the waiting time for a specialist consultation is usually decreased if there is a strong suspicion of RA. On the other hand, an imprecise or incorrect diagnosis in primary care delays the first appointment with the specialist consultant at the hospitals.

d) Difficulties in accessing biologics

The rheumatologists consider the approval of biologics prescription by the hospital a bureaucratic and controlled process. The patient medical history has to be scrutinized and authorization has to be obtained from several parties, including the department director, the prescribing specialist, the hospital pharmacy, the therapeutic commission and the Hospital Management Board, responsible for the final decision. This process may take from two days up to eight weeks. However, this complexity does not impact on the rheumatologist decision to prescribe biologics.

Moreover, rheumatologists noted that the criteria used for biologics prescription differ between the public and private setting, being more restrictive in the former.

According to the representative of ANDAR, most patients are unaware of the high added medical value of biologics and consequently they do not bring this subject into discussion during the consultation. This situation is further aggravated by the fact that patients, in general, have a passive relationship with physician leading to unilateral treatment decisions.

**Table 2:** Key barriers identified by stakeholder group and level of agreement

**Major research topic based on RA circuit**	**Key barriers identified**	**Level of agreement**						
		**U-GP**	**R-GP**	**REU**	**PHA**	**HMA**	**SPR**	**ANDAR**
		**(n = 5)**	**(n = 5)**	**(n = 9)**	**(n = 6)**	**(n = 4)**	**(n = 1)**	**(n = 1)**
Access to healthcare services	a) Difficulties in accessing primary healthcare services	0/5	3/5	NA	NA	NA	NA	NA
	b) Difficulties in RA diagnosis among GPs	0/5	1/5	9/9	NA	NA	1/1	1/1
	c) Difficulties in accessing secondary healthcare services	1/5	2/5	4/9	NA	NA	1/1	1/1
Treatment with biologics	d) Difficulties in accessing biologics	NA	NA	4/9	0/6	0/4	0/1	1/1

Level of agreement: number of stakeholders who recognised the issue as a key barrier in the RA circuit/ total sample interviewed among the group of stakeholders, n: total sample interviewed among the group of stakeholders, NA: research topic not addressed by this group of stakeholders, RA: rheumatoid arthritis, GPs: general practitioners, U-GP: urban general practitioner, R-GP: rural general practitioner, REU: rheumatologist, PHA: hospital pharmacy managers and purchasers, HMA: hospital managers; SPR: Portuguese Society of Rheumatology, ANDAR: Rheumatoid Arthritis Patient Association.

### Identification of leverage points

The leverage points to overcome the identified barriers to access treatment included the improvement of epidemiological and clinical knowledge about RA nationally, a better understanding of the disease by the patients and the GPs, the clarification of biologics benefits among budget holders and a raised awareness of the current treatment guidelines. Considering the identified leverage points, eight key initiatives were proposed regarding its attractiveness to stakeholders, its potential impact, the required investment and required effort (see Table 3).

**Table 3:** Leverage points and key initiatives identified by stakeholders

**Leverage points**	**Group of stakeholders**	**Key initiative**	Attractiveness for stakeholders^*^	**Potential impact**^**†**^	**Required investment**^**†**^	**Required effort**^**†**^
			**Global appreciation**			
Improve epidemiological and clinical knowledge of RA	U-GP, R-GP, REU, ANDAR	Optimization of RA National Registry	Clearly interesting	Medium	Medium	Medium
Promote patient’s knowledge about RA	U-GP, R-GP, REU, ANDAR	Dissemination of information on rheumatic symptoms in primary care centres	Clearly interesting	Medium	Low	Low
	U-GP, R-GP, REU, ANDAR	Valorisation of RA and adequate treatment	Likely interesting	Medium	Low	Low
Promote GPs’ knowledge about RA	U-GP, R-GP, REU	Regular visits of specialists to primary healthcare centres	Clearly interesting	Medium	Low	Medium
	U-GP, R-GP, REU, PHA	Regular training sessions about RA for GPs	Clearly interesting	Medium	Low	Low
Promote fulfilment of biologics guidelines	REU, SPR	Promote successful case reports about biologic treatment	Likely interesting	Medium	Low	Low
	REU, SPR	Promote broader utilization of diagnosis and monitoring tools	Likely interesting	Medium	Low	High
	HMA, REU	Implement hospital-based research to collect real-world data	Clearly interesting	Medium	Medium	Medium

Although barriers were identified, no leverage points resulted from the interviews with the budget holders.

*graded as: not interesting, likely interesting, clearly interesting. ^†^graded as: low, medium, high.

U-GP: urban general practitioner, R-GP: rural general practitioner, REU: rheumatologist, ANDAR: Rheumatoid Arthritis Patient Association, PHA: pharmaceutical managers and purchasers; SPR: Portuguese Society of Rheumatology, HMA: hospital managers.

## Discussion

By exploring the views of individuals who in Portugal represent the range of stakeholder groups involved in the RA therapeutic circuit in Portugal, this study identified the main factors limiting access to healthcare and treatment, and ultimately to biologics, and the key initiatives that may be undertaken to overcome these barriers. Qualitative research methods were useful for this purpose by collecting written descriptions of how individuals experience this research topic [15].

The most predominant view among stakeholders is that relevant barriers in RA are upstream of the rheumatology practice and consequently work needs to be done at the primary care level.

The lower density of GPs in rural areas as well as the unawareness of RA by the general population limits the access to primary care services in this clinical context. Access to rheumatologists is affected first by the geographical area of the patient, mainly in rural areas where hospitals are located outside the residential areas; second, by difficulties in diagnosing RA (which is often mistaken with other rheumatic diseases) at primary care level. These factors result in a longer and ineffective referral process to the specialist, leading to the delay of RA diagnosis and the postponement of the treatment with disease-modifying antirheumatic drugs (DMARDs) [11]. Many studies have shown that an early RA diagnosis and an aggressive management can limit the burden of the disease [9-11]. Furthermore, there is evidence that induction of remission early in the disease process can impact on its progression [7,16,17]. In Europe, efforts have been made to reduce time for RA diagnosis and treatment, by promoting early diagnosis [14]. However, because of different levels of disease awareness and diverse healthcare systems organisation, the success of these initiatives differs between countries.

At the secondary care level the most relevant barrier found was related to the difficulties in the approval of biologics prescription. These difficulties can be mainly explained by the high cost of biologics and the hard budget constraints observed in the Portuguese public hospitals.

We cannot ignore the possible existence of other factors limiting access to biologics at the hospital level. For example, although the lack of compliance to treatment guidelines was proactively explored during the interviews none of the relevant stakeholders viewed this as a key barrier to access to biologics.

Furthermore, none of the stakeholders believed there was a barrier directly influencing first-line treatment with non-biologic DMARDs (e.g. methotrexate) at the specialist setting.

The leverage points most valued by the interviewees at the primary care setting were focused on raising awareness about the disease among patients and GPs. This can be achieved by periodic and close interaction between rheumatologists and GPs, through regular visits of these specialists to primary care centres, disease workshops, clinical discussions and e-training or other similar activities. The analysis of successfully treated patients in the context of the clinical discussion will motivate GPs to diagnose and refer the patients to the specialist earlier. Clear and lean referral recommendations could also help GPs to identify cases requiring a specialist consultation.

Valorisation of RA among citizens must be enhanced and patients should be more aware of the RA symptoms. This could be achieved by awareness campaigns with the involvement of the media and through dissemination of information on rheumatic symptoms in primary care facilities. This sort of initiatives would have a considerable impact on the promotion of the disease among the general public.

The optimisation of the available registry of RA patients (Reuma.pt) [18] was a key initiative pointed out by the RA Patient's Association, GPs and rheumatologists, which aimed to improve knowledge about the disease pattern in Portugal. This online register has a national coverage, linking all public and private Rheumatology Departments. Since June 2008, rheumatologists have been collecting data on rheumatic patients receiving biologic therapies or synthetic DMARDs. However, interviewees noted that it is essential to professionalize this registry process to ensure the data is reliable and updated. This will certainly foster disease knowledge help monitor patient’s conditions in the secondary care setting. Currently nationwide epidemiological programs, EpiReumaPt [19] and CoReumaPt [20], are conducted to gather more epidemiologic data and to evaluate the burden of different rheumatic diseases in Portugal, including RA. The results of this program will increase the overall knowledge of RA and raise public awareness of the relevance of this condition and its impact on the Portuguese population. Ultimately, it is expected that all these activities increase patient’s access to treatment, which will contribute to a global improvement in RA healthcare.

At the specialist setting the leverage points most valued by the interviewed rheumatologists and HMA were focused on the promotion of the benefits of biologics and the fulfilment of specialists with treatment guidelines. The most attractive initiative for stakeholders relates with the implementation of hospital-based research to collect real-world data. It is widely accepted that well-designed observational studies can identify clinically important differences among therapeutic options and provide data on the long-term drug effectiveness and safety. This type of studies can complement findings from randomized controlled trials and therefore increase the clinician’s knowledge on the available treatments [21]. The interviewees believe that hospital-based studies comparing cohorts of RA patients using biologics versus other treatments would provide a clearer picture about the biologics effectiveness, safety profile and would allow a broader characterisation of the treatment patterns in the daily practice. A large electronic database, such as Reuma.pt, can be an invaluable resource for this type of research. A preliminary report from this registry was published in January 2011 characterizing a pool of 2,162 RA patients of which 700 were treated with biologics and 1,462 with synthetic DMARDs. This report characterised the average values of disease activity, the duration of treatment with biologics and the reasons for its discontinuation [18]. More recently, another study based on REUMA.pt made a comparative effectiveness analysis of predictors of response to tumour necrosis factor inhibitor therapies in RA [22]. This type of reports will help to clarify the effectiveness of biologics in the Portuguese clinical practice and will contribute to compliance with treatment guidelines.

Interestingly, two rheumatologists were apprehensive regarding the long term safety profile of biologics, particularly the potential increased risk of infection. This could suggest that concerns about hypothetical long-term safety issues rose in the initial phase of the use of biologics in the late 90’s have not been completely overcome by rheumatologists. However, this aspect was not considered a main barrier since only a minority of the interviewees saw it as a key factor within RA treatment. As long-term safety data for biologics are becoming available from large observational cohorts, such as those from national registries or from open label long-term extension of trials, it is reassuring to note that adverse effects tend to be more frequent in the early phase of treatment [23]. Infection risk remains the major concern, but it clearly peaks during the first months of use and decreases over time to relatively low levels. Malignancy was an initial concern, particularly for long-term therapy but in fact all available data is reassuring regarding this hypothetical issue [24].

The presentation of successful clinical cases related to the use of biologics was seen by specialists as a key initiative to promote its benefits and to demystify apprehensions about the safety of these agents. This initiative would require low investment and effort, involving the cooperation between healthcare centres and rheumatologists.

Rheumatologists believe that the use of RA evaluation tools such as 28-joint count Disease Activity Score (DAS28) should be encouraged. However, they recognise that this initiative can demand a high amount of effort, as it involves changing the established routine practices of some rheumatologists who may not follow this practice in patients who are treated with conventional DMARDs. DAS28 is an instrument to measure RA activity with a composite score of several variables including tenderness and swelling of 28-joint counts, erythrocyte sedimentation rate (ESR) and patient’s global assessment through a visual analogue scale [25]. DAS28 has been widely accepted as a valuable tool for measuring disease activity [26] and worldwide guidelines on RA management consider this parameter primordial. As in other countries, in Portugal the guidelines for the use of biologics in RA define specific disease activity levels (measured by DAS28) [27]. DAS28 would not only support rheumatologists in therapeutic decisions, but would also improve the fulfilment of specialists with the treatment guidelines. Therefore, from the specialist´s point of view, a wider use of DAS28 in clinical practice would contribute to a better management of the disease.

### Methodology discussion

We believe that the range of stakeholders groups selected in our study represents the most relevant and influential professional areas involved in the therapeutic circuit of RA in Portugal. The number of interviews per stakeholder group was adequate since data overall saturation method was followed. In addition, our study was conducted within the most representative geographical areas. Still, the perspective of the patient was not explored in this research. Patients are important stakeholders in a therapeutic circuit and their involvement would certainly enlighten our findings [28]. Nonetheless, the Portuguese RA patient’s association (ANDAR) has a large number of associates and systematically collects their views, attitudes towards different problems, including those linked to treatment. We believe that by exploring the views of a representative from this association we obtained a reliable overview of RA patients’ experiences under this framework.

Although qualified and trained researchers were involved, we cannot ignore the increased risk of interviewer bias inherent to semi-structured interviews (e.g. prescriptive or leading questions).

The use of data-triangulation allowed the validation of the findings presented and conclusions were consolidated by a final meeting with one representative of each stakeholder group.

None the stakeholders involved had any vested interest in the project, thus biased views can be excluded.

## Conclusions

The most predominant view emerged from this qualitative study is that the most important barriers conditioning access to treatment by RA Portuguese patients, and in particular to biologics, are upstream of rheumatology practice. Actions should be implemented at the primary care level to improve referral to rheumatologists and, consequently promote the access to an adequate treatment. This can be achieved by disseminating knowledge about the disease among GPs and patients, by providing the former with periodic and close interaction with rheumatologists and the later with more information on RA symptoms and the advantages of adequate treatment. Long-term observational studies collecting daily clinical activity data are essential to characterize the local RA population, to improve disease management and to increase compliance with the current treatment guidelines. Increased communication and interaction between stakeholders in the RA treatment framework is crucial to overcome existing barriers.

## Competing interests

The authors declare that they have no competing interests.

## Authors’ contributions

All authors critically revised the manuscript for important intellectual content and approved the final manuscript. PAL and RM: Made substantial contributions to the conception and design of this research, and were involved in the interpretation of data and drafting of the manuscript LV, AMP and RC: were involved in the interpretation of data, drafting and revision of the manuscript. JEF: Was involved in the interpretation of data, critical revision of the manuscript and oversight of the submission process.

## Authors’ information

PAL, MSc, has recently published a paper in the European Journal of Health Economics (Epub ahead of print) entitled “Patients’ access to biologics in rheumatoid arthritis: a comparison between Portugal and other European countries”. Although from a different perspective, the current qualitative research complements this former publication and brings new insights regarding the reasons undermining the observed differences between PT and other EU countries.

JEF, MD, PhD is the Head of the Rheumatology Research Unit and of the Biobank at Instituto de Medicina Molecular, Faculdade de Medicina da Universidade de Lisboa. He is Assistant Professor of Rheumatology and a Rheumatology Consultant at the Lisbon Academic Medical Centre. He is also the President-Elect of the Portuguese Society of Rheumatology.

## Pre-publication history

The pre-publication history for this paper can be accessed here:

http://www.biomedcentral.com/1471-2474/14/279/prepub

## Supplementary Material

Additional file 1 **Complete set of questions addressed by the stakeholders.** List of questions of the semi-structured interviews, tailored to the background of the stakeholders. Click here for file
