# Supplementary material for: Patient’s access to healthcare and treatment in rheumatoid arthritis: the views of stakeholders in Portugal
Source: BMC Musculoskelet Disord. 2013 Sep 25;14:279. doi: 10.1186/1471-2474-14-279 (PMC3849024; doi:10.1186/1471-2474-14-279)
Supplement: Additional file 1 — Complete set of questions addressed by the stakeholders. List of questions of the semi-structured interviews, tailored to the background of the stakeholders. [file 1471-2474-14-279-S1.docx]

| **Major topic** | **#** | **Questions** |
| --- | --- | --- |
| Access to healthcare services | 1 | What are the main difficulties regarding the accessibility to primary healthcare services? |
|  | 2 | What are the signs and symptoms that may alert the physician for a possible diagnosis of RA? |
|  | 3 | Upon suspicion of RA which diagnostic tests/exams are performed? |
|  | 4 | Do you think the reimbursement of anti-CCP would be a useful measure to facilitate the diagnosis? |
|  | 5 | Is the RA diagnosis easy to establish or is the disease easily mistaken for others? What difficulties may be found in the disease diagnosis? |
|  | 6 | What are the main causes associated with late diagnosis/no diagnosis of the disease? |
|  | 7 | What are the reasons behind the late RA diagnosis in primary healthcare services? |
|  | 8 | Are all patients diagnosed with RA referred to secondary healthcare services, or some may be followed in primary healthcare? |
|  | 9 | In which circumstances is a patient referred from primary healthcare services to specialty consultations? Is the referral flow easy or are there many refusals? |
|  | 10 | How would you describe the liaison between primary healthcare services and specialists? |
|  | 11 | In average, how long patients wait for a specialty consultation? |
|  | 12 | According to your perception are there a large number of patients attending private consultations? Do you have an estimate for this proportion? |
|  | 13 | Is the information usually included in the referral report enough for the screening by the specialist? |
|  | 14 | Does the patient attend to the hospital specialty with the correct diagnosis? What’s the proportion of misdiagnosed patients? |
|  | 15 | Do the general practitioners have enough background information to perform an accurate diagnosis? |
|  | 16 | Where do RA patients who attend to the hospital come from? (emergency services / primary healthcare services / other) |
|  | 17 | What is the proportion of RA patients who attend to the hospital with moderate or severe disease? |
|  | 18 | In your opinion, what is the proportion of undiagnosed patients? Why? |
|  | 19 | Are there any country regions with a larger casuistry of RA? Which ones? |
|  | 20 | How would you describe the patients’ level of knowledge of their disease and available medications? |
|  | 21 | Are there any awareness campaigns aiming to alert the patients about the disease symptoms and treatments? Are these (or would these) be efficient? |
|  | 22 | Are there any regular training actions for physicians (primary healthcare and specialists) about the disease and therapeutic alternatives? Are these actions sufficient? |
|  | 23 | Do specialists regularly share their cases/questions with each other? Would it be interesting to promote the exchange of information, namely through the use of web-based platforms? |

Appendix A Complete set of questions addressed by the stakeholders

| Rheumatoid arthritis treatment access | 24 | Which RA treatment guidelines do you know/follow? Which topics are foreseen in these guidelines? |
| --- | --- | --- |
|  | 25 | According to your perception, are these guidelines generally followed by the physicians? |
|  | 26 | Do you use the DAS28 in your patients? |
|  | 27 | How do you monitor your RA patients? |
|  | 28 | What actions could be implemented to include the DAS28 in clinical practice? Are more training actions needed? |
|  | 29 | What is the percentage of newly diagnosed patients that attend your consultations? |
|  | 30 | What is the proportion of your RA consultations? |
|  | 31 | How often is the disease progression assessed? Guidelines recommend assessment every 3 months. Is this frequency achievable? |
|  | 32 | In average, how many specialty consultations are performed each year, for a patient with RA? How many should be performed? |
|  | 33 | Is it easy to ascertain if the patient has mild, moderate or severe RA? Is the staging performed in accordance with the guidelines (DAS28)? |
|  | 34 | Which factors determine the therapeutic decision? Is the severity of the disease one of such factors? How are the more advanced stages of the disease managed? |
|  | 35 | Which types of medication are prescribed in primary healthcare services for RA? |
|  | 36 | What is the usual prescription sequence, considering the type of medication [non-steroidal anti-inflammatory drugs, methotrexate/azathioprine and biologic treatments (combined and not)]? How long is the patient expected to remain on each type of medication? |
| Treatment with biologics | 37 | What is the usual prescription and administration procedure for biologic treatments in the hospital setting? An approval is required from the Department Director, the Pharmacy Committee or other? |
|  | 38 | Which warning signs may indicate the need for a change of therapy? |
|  | 39 | In your daily practice, do you prescribe biologic treatments? If not, why? |
|  | 40 | Which conditions determine the initiation, maintenance and discontinuation of the biologic treatment? |
|  | 41 | When is the biologic treatment prescribed as first line therapy? |
|  | 42 | What type of information do you receive regarding biologic products? And about other treatments for this disease? What’s the source of this information? How often do you receive it? |

| Hospital budget management | 43 | Is the hospital pharmacy’s budget cap for medication dispensing usually met or surpassed? What measures are implemented if the budget is surpassed? |
| --- | --- | --- |
|  | 44 | Is this budget spread by medication groups? |
|  | 45 | Is the medication prescribed outside the hospital and supplied by the hospital pharmacy also reimbursed within this budget cap? |
|  | 46 | How are free provisions of drugs recorded?  For all biologics purchasing costs that are totally reimbursed by Health Authorities, how is it accounted in terms of hospital drug expenditure? |
|  | 47 | Are these drugs reimbursed by the Regional Health Administrations (ARS)? |
|  | 48 | Which are the control measures for drugs freely provided by the hospital pharmacy for prescriptions outside the hospital? |
|  | 49 | Is there any control of prescriptions in the public hospital *versus* private practice? |
|  | 50 | Does the hospital have specific protocols for RA? |
|  | 51 | Considering the heavy burden of biologic drugs to the total drug costs supported by the hospitals and NHS, how could the access to these products be managed, considering the budget constraints? |
|  | 52 | Which is the policy followed by the hospital for the purchasing of biologic drugs? Are one or many vendors preferable? |

#: question number, RA: rheumatoid arthritis. The questions were tailored to the stakeholder’s background. The questions 12, 14, 17, 18 and 29 were also scoped under the major topic “Estimation of target RA patients”
